# West Nile Virus Detection and Commercial Assays

**DOI:** 10.3201/eid1107.041149

**Published:** 2005-07

**Authors:** Peter A.G. Tilley, Gail A. Zachary, Roberta Walle, Paul F. Schnee

**Affiliations:** * Provincial Laboratory for Public Health, Calgary, Alberta, Canada;; † Palliser Health Region, Medicine Hat, Alberta, Canada

**Keywords:** West Nile Virus, IgM, West Nile Fever

**To the Editor:** Roehrig and colleagues described the long-term persistence of immunoglobulin (Ig) M antibody in patients with West Nile virus (WNV) infection, as tested using an in-house Centers for Disease Control and Prevention (CDC) enzyme immunoassay (EIA) (1). This result suggests that interpreting WNV IgM results in subsequent years would be difficult. With the commercial availability and widespread use of US Food and Drug Administration–approved WNV IgM tests, we were concerned that this phenomenon might also occur with new tests. Thus in 2004, we initiated a follow-up study of patients infected during the inaugural (2003) WNV season in Alberta, Canada.

Fifty patients who were WNV IgM positive by 2 commercial IgM kits (West Nile virus capture EIAs, Focus Technologies, Cypress, CA, USA, and Panbio, Windsor, Queensland, Australia) during the fall of 2003 were contacted. Sera were recollected and tested for IgM and IgG antibodies to WNV with current kits from these 2 companies. Sera were also tested for hemagglutination-inhibiting (HI) antibodies to WNV (2).

Of 39 serum samples from 38 patients, 28 were positive, 5 were indeterminate, and 6 were negative with the Focus IgM kit. Twenty-one were positive, 3 were indeterminate, and 14 were negative with the Panbio IgM kit. All had WNV IgG antibodies detected by Focus and Panbio IgG kits. We detected HI antibodies to WNV in all patients, and titers in 12 were ≥320. The time course for IgM index values for the Focus IgM kit used in 2003 and 2004 is shown in the Figure.

**Figure:**
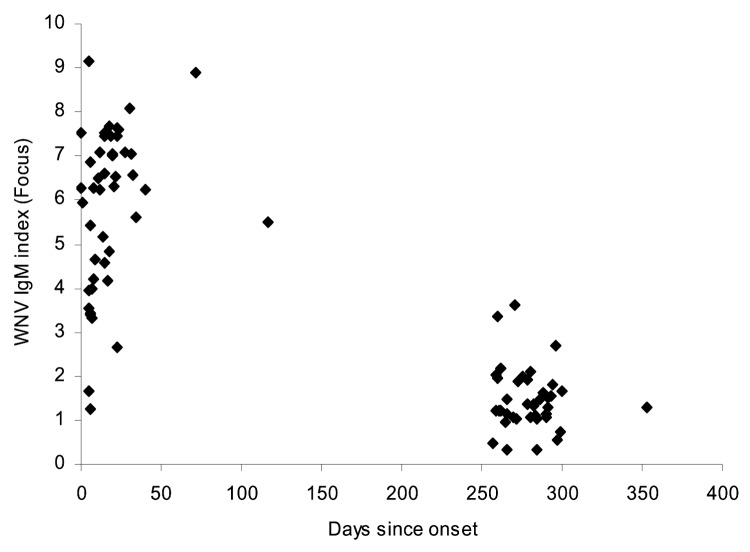
West Nile virus (WNV) immunoglobulin (Ig)M index values in serum specimens from 38 WNV case-patients detected in the fall of 2003. The assay was performed by using the Focus Technologies kit, as per the manufacturer's instructions. An index >1.1 indicates a positive result and an index <0.9 indicates a negative result.

These data show that when tests are conducted with newly available kits, as with the CDC in-house test, IgM antibody to WNV persists for ≥8 months in most patients. A single high HI titer is not helpful in identifying recent infection. In addition, the IgM test cannot differentiate between recent and past infections. Interpreting a positive IgM result in WNV-endemic areas will be complex because a positive WNV IgM result could indicate a current acute infection or a previous WNV infection even in a person with a different acute illness.
